# Current Prognostic and Predictive Biomarkers for Endometrial Cancer in Clinical Practice: Recommendations/Proposal from the Italian Study Group

**DOI:** 10.3389/fonc.2022.805613

**Published:** 2022-04-08

**Authors:** Gian Franco Zannoni, Emma Bragantini, Francesca Castiglione, Matteo Fassan, Giancarlo Troncone, Frediano Inzani, Anna Pesci, Angela Santoro, Filippo Fraggetta

**Affiliations:** ^1^ Unità di Ginecopatologia e Patologia Mammaria, Dipartimento Scienze della Salute della Donna, del Bambino e di Sanità Pubblica, Fondazione Policlinico Universitario A. Gemelli Istituto di Ricerca e Cura a Carattere Scientifico (IRCCS), Rome, Italy; ^2^ Istituto di Anatomia Patologica, Università Cattolica del Sacro Cuore, Rome, Italy; ^3^ Department of Surgical Pathology, Ospedale S. Chiara, Trento, Italy; ^4^ Histopathology and Molecular Diagnostics, Careggi University Hospital, Florence, Italy; ^5^ Department of Medicine - DIMED, University of Padova, Padova, Italy; ^6^ Department of Public Health, University of Naples Federico II, Naples, Italy; ^7^ Department of Pathology, Sacred Heart Hospital Don Calabria Negrar, Verona, Italy; ^8^ Pathology Unit, “Cannizzaro” Hospital, Catania, Italy; ^9^ Pathology Unit, “Gravina” Hospital, Caltagirone, Italy

**Keywords:** endometrial cancer, prognostic markers, histomolecular classification, pathological guidelines, prognostic stratification

## Abstract

Endometrial carcinoma (EC) is the most common gynecological malignant disease in high-income countries, such as European countries and the USA. The 2020 edition of the World Health Organization (WHO) Classification of Tumors of the Female Genital Tract underlines the important clinical implications of the proposed new histomolecular classification system for ECs. In view of the substantial genetic and morphological heterogeneity in ECs, both classical pthological parameters and molecular classifiers have to be integrated in the pathology report. This review will focus on the most commonly adopted immunohistochemical and molecular biomarkers in daily clinical characterization of EC, referring to the most recent published recommendations, guidelines, and expert opinions.

## Introduction

Endometrial carcinoma (EC) is the most common gynecological malignancy in Western countries. It represents the sixth most commonly diagnosed cancer and the 14th leading cause of cancer death in women worldwide (1). The 5th edition of the WHO of Tumors of the Female Genital Tract, published in 2020, considers the molecular classification system for ECs as an additional tool to integrate traditional histopathological criteria [histological type and grade, pattern of myometrial involvement, lymphovascular involvement (LVSI) and FIGO stage] (2). The aim of this integrated histomolecular approach is to unify both classical pathological parameters and molecular data in one pathology report and correlate them to the clinical risk groups, for a better multidisciplinary definition of patient outcomes (3).

## Aim

**Figure 1:**
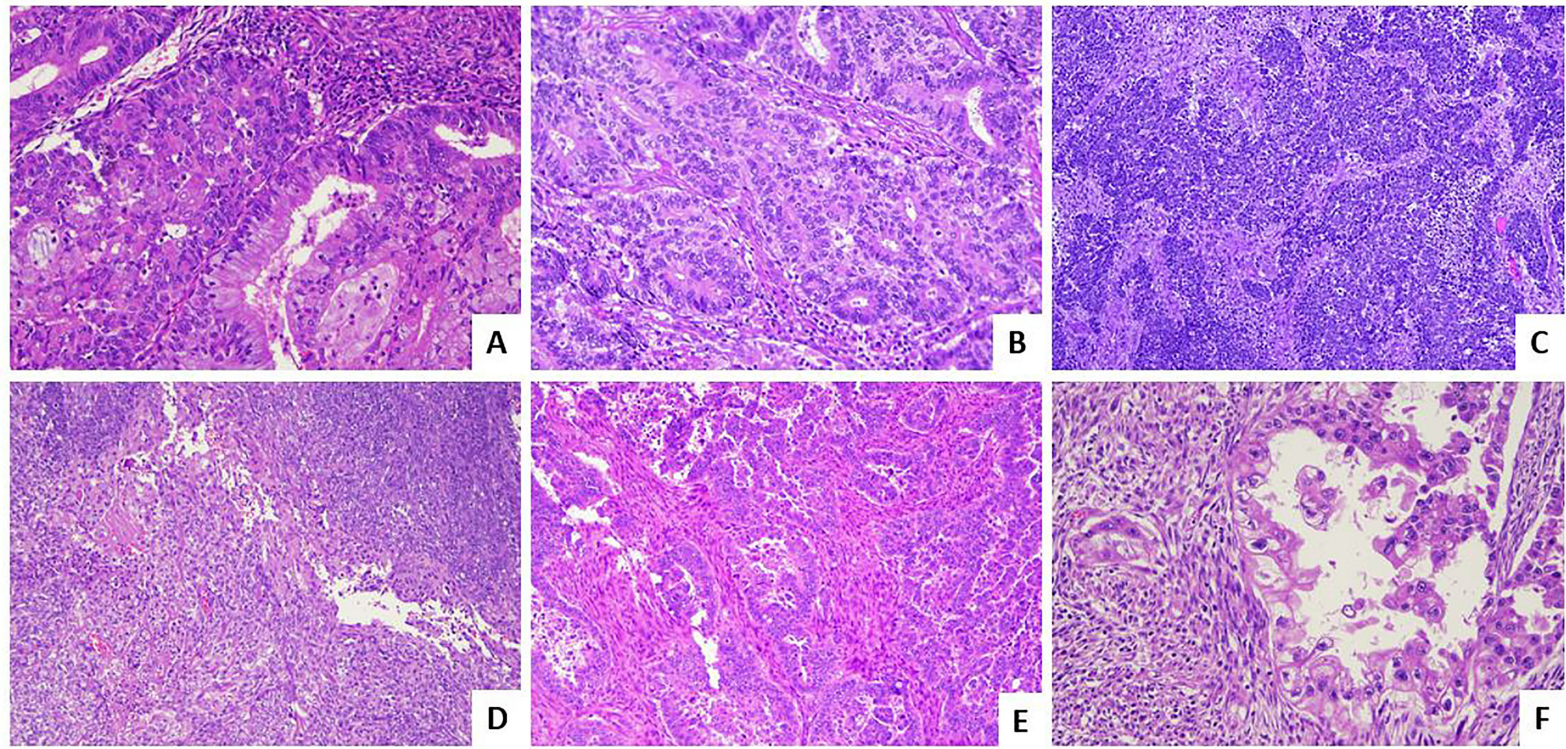
Histological subtypes of endometrial carcinoma: an overview. **(A)** An endometrioid carcinoma G1 FIGO with mucinous features (LSAB, 10×). **(B)** An endometrioid carcinoma G2 FIGO (LSAB, 10×). **(C)** An endometrioid carcinoma G3 FIGO with basaloid features (LSAB, 4×). **(D)** An endometrioid carcinoma G3 FIGO with spindle cell features (LSAB, 4×). **(E)** A serous carcinoma (LSAB, 10×). **(F)** A clear cell carcinoma (LSAB, 20×).

This overview will focus to enlighten and summarize the histological features (**Figure 1**) and the immunohistochemical and molecular biomarkers useful to perform a right and complete characterization of endometrial cancer.

**Figure 2:**
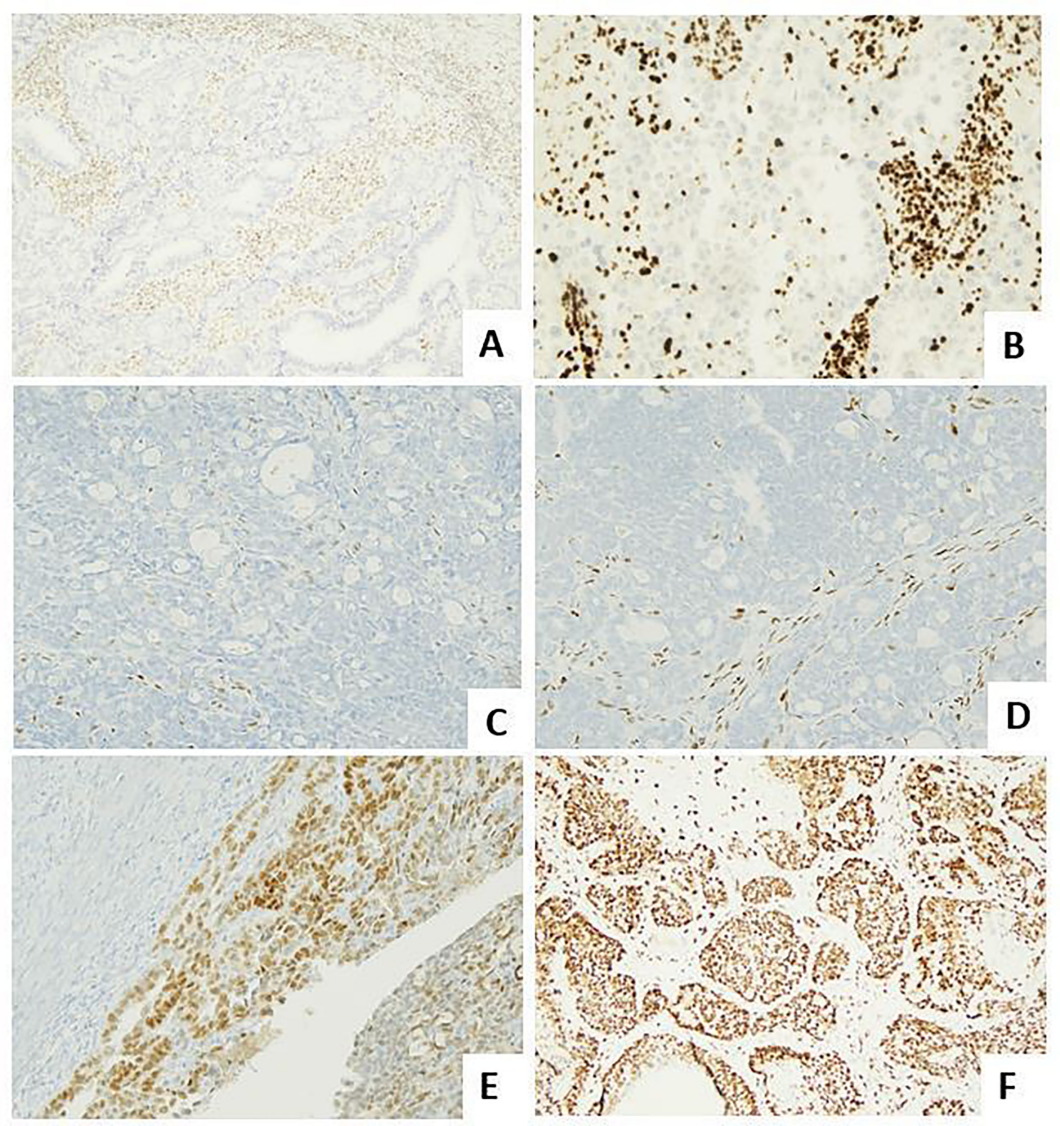
Molecular landscape of endometrial carcinoma: the role of the immunohistochemical surrogates. **(A)** An endometrioid carcinoma G2 FIGO with loss of MLH1 expression (LSAB, 10×). **(B)** An endometrioid carcinoma G2 FIGO with loss of MSH2 expression (LSAB, 10×). **(C, D)** An endometrioid carcinoma G3 FIGO with loss of ER (LSAB, 10×) and PR (LSAB, 10×). **(E)** An endometrioid carcinoma G2 FIGO with expression of ER in 80% of neoplastic cells, with a 2+ score intensity (LSAB, 10×). **(F)** A serous carcinoma with overexpressed aberrant pattern of p53 (LSAB, 4×).

**Figure 3:**
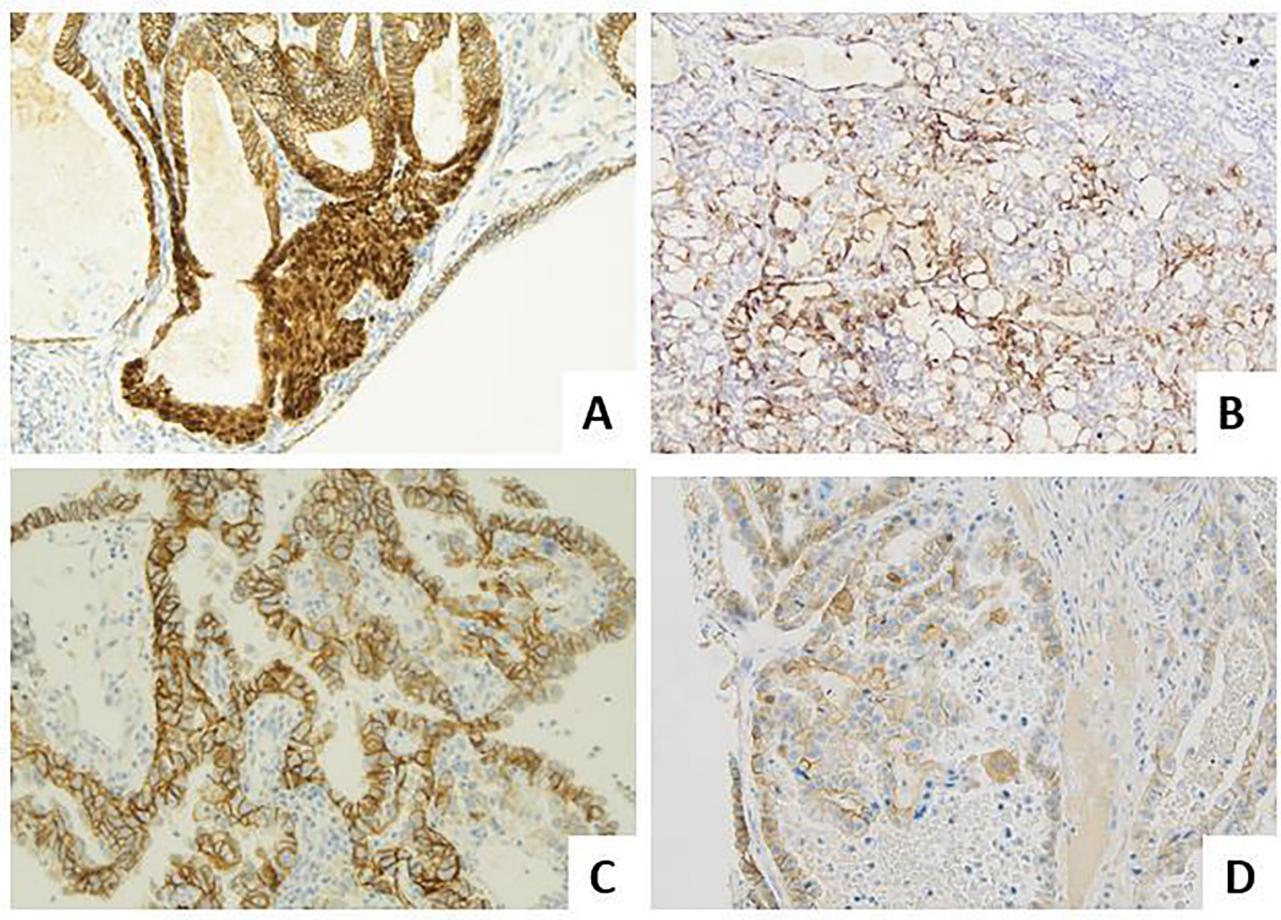
Molecular landscape of endometrial carcinoma: the role of other immunohistochemical markers. **(A)** An endometrioid carcinoma G1 FIGO with nuclear beta-catenin expression in foci of morular metaplasia (LSAB, 20×). **(B)** An endometrioid carcinoma G2 FIGO with L1CAM over-expression (>10%) (LSAB, 4×). **(C)** A serous carcinoma with HER2 score 3+, according to the 2007 modified ASCO CAP scoring method (LSAB, 20×). **(D)** A clear cell carcinoma with HER2 score 2+, according to the 2007 modified ASCO CAP scoring method (LSAB, 20×).

Considering the important insights into the biological characterization and clinical management of EC, the Italian Society of Pathological Anatomy and Cytopathology (SIAPEC) and members of PAGINE (Italian Group of Gynaecological Pathology) worked on a collaborative project to draft consensus specific recommendations on the (i) most important definitions related to the 2013 The Cancer Genome Atlas (TGCA) histomolecular classifications; (ii) methods of POLE, MSI, p53 testing on cancer tissue (**Figure 2**); and (iii) methods of other (optional) prognostic marker evaluation on cancer tissue (**Figure 3** and **Tables 1**-**8**). Members of the working group have been selected among professionals with high standard records on scientific activity and routine pathological and clinical work on gynecological pathology and molecular testing of cancers, comprising 10 pathologists/molecular pathologists.

**Table 1:** POLE: recommendations and comments from the working group.

Recommendations
A) No surrogate for POLE mutation still exists but the targeted sequencing for the common mutations in this gene could be used rather than whole genome or panel testing (mutation analysis of the exonuclease domain of POLE exons 9, 11, 13, and 14).
Comments
The mutational analysis of the exonuclease domain of POLE should be considered in the following cases:
- EEC G3 and other high-grade histologies (UEC/DEC, clear cell, carcinosarcoma) - rare histotypes (neuroendocrine tumors) - abundance in TIL and/or peritumoral lymphocytes - mixed cases - ambiguous morphologies - ambiguous immunophenotype (possible multiple classifiers) - subclonal p53 at IHC
Mutational analysis should be carried out only in selected experienced centers.
Minimal requirement is the adequate assessment of the 5 more frequent occurring POLE hotspot variants. Unknown variants or VUS should be discussed at the Tumor Board.

**Table 2:** MSI: recommendations and comments from the working group.

Recommendations
A) The first method for MSI testing is represented by MMR IHC, a widely available laboratory test, utilizing antibodies against MLH1, MSH2, MSH6, and PMS2. B) MSI-PCR-based molecular testing is indicated in case of indeterminate IHC results (disagreement or interpretative difficulties). The five poly-A panel is the recommended panel given its higher sensitivity and specificity. C) As a novel alternative tool for MSI testing, NGS should be carried out only in selected centers experienced in these techniques.
Comments
- Use the IHC approach for detecting the four MMR proteins and assessing MMRd in any sporadic cancer type belonging to the spectrum of cancers found in Lynch Syndrome, so including EC. - Standardize pre-analytical and analytical protocol of testing - IHC can be performed on biopsies or surgical specimens if available, preferring the best-preserved sample as first choice
The main advantages of performing IHC on biopsies are the following:
(i) the better degree of fixation of biopsies (ii) the early knowledge of MSI status in a pre-operative setting The main advantages of performing IHC on surgical samples are the following: (i) larger amount of tumoral representative tissue; (ii) the possibility to select the best specimen for IHC testing; (iii) the possibility to overcome tumor heterogeneity. - The presence of an internal positive control is mandatory for interpretation of results. - Move to MSI-PCR or NGS (in selected centers) as a confirmatory test or whenever there is any doubt in IHC interpretation. In particular, in the following events: - Indeterminate/equivocal/ambiguous IHC results - False-negative MMR immunostainings mainly caused by pre-analytical poor tissue fixation - Aberrant staining patterns such as cytoplasmic, dot-like, or perinuclear staining – Loss of only one heterodimer subunit (e.g., only MLH1 or only PMS2 and not both)

**Table 3:** P53: recommendations and comments from the working group.

Recommendations
A) The first method for p53 testing is represented by IHC, a widely available laboratory test, utilizing antibodies against p53. B) Genetic testing (Sanger sequencing or NGS) is indicated in case of indeterminate IHC results (disagreement or interpretative difficulties). C) Genetic testing should be carried out only in selected centers experienced in these techniques.
Comments
- Use the IHC approach for detecting p53 pattern of expression - Standardize pre-analytical and analytical protocol of testing - IHC can be performed on biopsies or surgical specimens if available, preferring the best-preserved sample as first choice
The main advantages of performing IHC on biopsies are the following:
(i) the better degree of fixation of biopsies (ii) the early knowledge of p53 status in a pre-operative setting The main advantages of performing IHC on surgical sample are the following: (i) larger amount of tumoral representative tissue; (ii) the possibility to select the best specimen for IHC testing; (iii) the possibility to overcome tumor heterogeneity. - The presence of an internal positive control is mandatory for interpretation of results. - Move to genetic testing as a confirmatory test or whenever there is any doubt in IHC interpretation.
We retain that undoubtedly diagnostic interpretative challenges by p53 immunohistochemistry are possible events, but we are also aware that equally there are similar issues around quality assurance for genetic testing of TP53 mutation (Sanger sequencing or NGS) that may not be totally reliable.

**Table 4:** Hormonal receptors: comments from the working group.

Comments
- ER and PR are affordable immunohistochemical markers, available in all pathological laboratories worldwide and thus this scoring system could be easily implemented in routine practice. - Scoring for ER and PR adds relevant prognostic information to current clinical practice - Correlation between ER/PR expression in preoperative and postoperative material could be investigated also in EC, as well as already test in breast cancer - Reporting of percentages in the form of continuous or semicontinuous values for ER/PR expression, avoiding dichotomous values (e.g., positive, negative) is recommended - Reporting of intensity score is also advised - Immunohistochemical analysis for ER and PR expression can be performed manually or by digital image analysis that could provide a more objective and reproducible evaluation

**Table 5:** L1CAM: comment from the working group.

Comment
Similarly to beta-catenin, since ESGO/ESTRO/ESP recent guidelines do not consider L1CAM in EC molecular risk stratification, but considering that L1CAM could represent an additional marker to personalize patient treatment, we propose to introduce the use of L1CAM only with a scientific intent.

**Table 6:** Beta-catenin: comment from the working group.

Comment
Since there is still scientific debate to introduce the use of beta-catenin immunohistochemistry in the prognostic stratification of EC, we propose to analyze it only with a research purpose.

**Table 7:** PDL1: comments from the working group.

Comments
- To date, immunohistochemistry is the gold standard for PD-L1 expression assessment - Pathologists should be aware that this analysis is significantly affected by several factors: (i) different standardization protocols of PD-L1 assays (ii) different immunohistochemical commercially available antibody clones used among the different institutions (iii) different scoring algorithms evaluating PD-L1 positivity in tumor cells (TCs) and/or in immune cells (ICs) separately or in combination (combined positive score, CPS) (iv) use of different cutoffs (v) possible intratumoral heterogeneity of PD-L1 expression - An adequate immunohistochemical PDL1 evaluation should consider positive (lung macrophages, placental, spleen, and tonsil) and negative controls (alveolar cells, hepatocytes, and normal squamous epithelium) - In particular, in EC, it seems that CPS has shown methodological advantages over cell type-specific scoring systems (TPS and ICS)

**Table 8:** HER2: comments from the working group.

Comments
- Consider HER2 testing in advanced/recurrent endometrial serous carcinoma and in the mixed forms with serous component - In mixed carcinomas, HER2 testing should be performed on a tissue block containing the highest amount of serous component - For other high-grade histologies, we propose to introduce HER2 testing only with a scientific intent - The first method for HER2 testing is represented by HER2 IHC - Molecular reflex test (FISH/SISH) is indicated in case of equivocal (2+) IHC results - Standardize pre-analytical and analytical protocol of testing - IHC can be performed on biopsies or surgical specimens if available, preferring the best-preserved sample as first choice	
The main advantages of performing IHC on biopsies are the following:	
(i) the better degree of fixation of biopsies (ii) the early knowledge of HER2 status in a pre-operative setting	
The main advantages of performing IHC on surgical sample are the following:	
(i) larger amount of tumoral representative tissue (ii) the possibility to select the best specimen for IHC testing (iii) the possibility to overcome tumor heterogeneity	

## From 2013 TCGA Revolution to the Molecular Surrogates for EC Biological Characterization

In 2013, TCGA Research Network reported a large-scale, integrated genomic, transcriptomic, and proteomic analysis of 373 ECs, including 307 endometrial endometrioid carcinomas (EECs), 53 serous endometrial carcinomas (SECs), and 13 mixed cases, showing that EC could be stratified into four prognostically relevant molecular groups: POLE/ultramutated (7%) with excellent prognosis with no recurrence regardless of the FIGO grade, the microsatellite-instability/hypermutated (28%) and the copy-number-low/endometrioid (39%) characterized by intermediate prognosis, and the copy-number-high/serous like (26%) characterized by SECs and showing poor prognosis (4).

As the molecular classification proposed by the TCGA used cost prohibitive methods for group assignment in routine clinical practice, subsequent studies, in particular the Leiden/PORTEC group and the Vancouver/ProMisE group, developed and validated a molecular surrogates based on immunohistochemistry expression of p53 protein and mismatch repair (MMR) proteins and POLE sequencing. The four groups identified with such approach, i.e., POLE-mutant (POLEmut), MMR-deficient (dMMR), p53-abnormal (p53abn), and no specific molecular profile (NSMP), have been recently integrated in the European guidelines for endometrial cancer management (5, 6) with important clinical implications as the following:

- all POLEmut carcinomas up to FIGO stage II are included in the low-risk group and can be managed by observation alone
- p53mut EECs are lumped together with non-endometrioid carcinomas in the high-risk group and, in the absence of myometrial invasion, are considered at intermediate risk (7).

## POLE Subgroup

### Clinical Definition and Therapeutical Implications

The POLE1–4 genes encode for one of the four subunits that form Polϵ (DNA polymerase epsilon). POLE gene contains both the catalytic active site and the proofreading exonuclease domain (8). Polϵ is a protein responsible for the polymerization of the leading strand during DNA replication. It also possesses 3’ to 5’ exonuclease capability to repair misincorporated nucleotides during DNA replication and it is also involved in DNA repair pathways such as MMR, base excision repair (BER), nucleotide excision repair (NER), or double-strand break repair (9).

A few missense germline mutations in the proofreading domain of POLE affecting the exonuclease repair of Polϵ have been shown to be pathogenic such as W347C, N363K, D368V, L424V, P436S, or Y458F. These mutations hence result in a mutation rate increase of about 100-fold. Accordingly, these tumors are usually called ultramutated (10).

Pathogenic somatic mutations in the proofreading domain of POLE have been found in some tumor types, such as endometrial tumors (8%) and at lower frequencies in colorectal, glioblastoma, ovary, prostate, breast, or gastric cancer (9, 11–14). These mutations seem to confer similar phenotypes regardless of the tumor tissue type. These are missense, heterozygous mutations where no second hit by either mutation or LOH seems to be required, and they are very early events, possibly initiating. Some mutations are hotspots such as P286R, S297F, V411L, or S459F, but other rarer mutations have also been identified (e.g., P286H/L, S297Y, F367S, L424V/I, P436R, M444K, and A456P). These mutations result in ultramutation with an over-representation of C>A (15). Moreover, POLE tumors are hardly ever concomitant with MSI, although a few tumors with both phenotypes have been described, but they do not seem to show chromosomal instability as their karyotype is nearly diploid (16). Patients with somatic POLE driver mutations are younger on average and the prognosis is excellent in the early stage (15).

In EC, the POLE mt group is characterized by somatic mutations in the exonuclease domain of POLE, which encodes the catalytic subunit of DNA polymerase epsilon. Ultramutated tumors have an extraordinarily high mutation rate (232 × 10^−6^ mutations per megabase), included both low-grade (uncommonly) and high-grade EECs (significantly) and consistently showed the most favorable prognosis with no recurrence and this excellent outcome appears to be maintained across the several histotypes, regardless of the FIGO grade and other clinicopathological factors (17). According to the recent ESGO/ESTRO/ESP guidelines, all POLEmut carcinomas up to FIGO stage II, regardless of FIGO grade, histotype, or LVSI, are included in the low-risk group and could be managed by observation alone, with no need for adjuvant treatment (7).

For the very high immunogenicity (abundance of pre-existing tumor-antigen-restricted CD8+T-cells), with upregulation of immune checkpoint and other immunosuppressive genes, the POLEmut ECs together with MSI ECs, as “hot tumors”, are promising candidates for checkpoint blockade immunotherapy, being considered the best molecular types that can respond to anti-PD-1/PDL1 treatment (16). They may also be sensitive to treatment with nucleoside analogs as they increase the mutation burden to a level where tumor cells are not viable.

### Pathological Features

Typical histopathological features of a POLE mut ECs are as follows:

- endometrioid histotype
- high grade, with foci demonstrating severe nuclear atypia and giant cells
- high mitotic index
- abundance in tumor-infiltrating lymphocytes (TILs) and/or peritumoral lymphocytes
- morphological heterogeneity and ambiguity
- substantial LVSI
- subclonal p53 at immunohistochemistry (IHC)

### Interpretation Issues

Currently, there is no surrogate markers for POLE mutation and targeted sequencing for the common mutations or whole genome or panel testing must be used (mutation analysis of the exonuclease domain of POLE exons 9, 11, 13, 14). Not all POLE mutations impact prognosis as nearly all (>95%) POLE mutations outside the exonuclease domain are not associated with a ultramutated phenotype, and part of the mutations inside the exonuclease domain are not pathogenetic (18). For the five most common POLE mutations (P286R, V411L, S297F, A456P, and S459F), pathogenicity (in this sense meaning causal for tumor ultramutation) has been confirmed (18); however, the classification of other, less frequent POLE variants is currently challenging and variants of unknown significance (VUS) include the following: A465V, L424V, T278M, and A428T (18).

## Defective DNA Mismatch Repair Complex as Surrogate of Microsatellite Instability

### Clinical Definition and Therapeutical Implications

MMR is a highly conserved protein complex for recognizing and repairing erroneous short insertion, short deletion, and misincorporation/mismatches of bases that can arise during DNA replication and recombination, as well as repairing some forms of DNA damage. The most important MMR players include MLH1 (mutL homologue 1), MSH2 (mutS homologue 2), MSH6 (mutS homologue 6), and PMS2 (postmeiotic segregation increased) (19).

These four proteins function in heterodimers: MLH1-PMS2 and MSH2-MSH6 43,44 (where MLH1 and MSH2 are obligatory partners of these heterodimers) (20, 21). An alteration in MLH1 and MSH2 results in subsequent proteolytic degradation of the mutated protein and its secondary partner (21). Conversely, mutations in secondary PMS2 or MSH6 may not result in proteolytic degradation of their primary partners, as MSH6 can be substituted in the heterodimer by MSH3, and PMS2 by PMS1 or MLH3. Consequently, the PMS2 antibody detects all cases that harbor either MLH1 or PMS2 abnormalities (22), and the MSH6 antibody detects all cases that harbor either MSH2 or MSH6 abnormality. MLH1 and MSH2 alone do not recognize cases with PMS2 or MSH6 abnormalities.

The inactivation of these genes (i.e., DMMR) can occur due to germline and/or somatic mutations or epigenetic silencing, resulting in the accumulation of frame-shift mutations (either through insertions or deletions) with a subsequent increased mutational burden, especially within repetitive stretches of DNA, called microsatellites (short tandem repeats, generally of a sequence that ranges from one to six bases), being distributed along the genome of both coding and non-coding regions and particularly sensitive to DNA mismatching errors during DNA replication or iatrogenic damage; this manifests as microsatellite instability (MSI). Thus, MSI is a condition of genetic hypermutability resulting from defective DNA MMR, and the two terms are often used interchangeably (22).

Germline mutation(s) of the MMR genes is the hallmark of constitutional mismatch repair deficiency (CMMRD) (23). CMMRD is due to a biallelic MMR gene mutation in which MMR defects are inherited from both parents. Mutations occur in well-characterized MMR genes including MLH1, PMS2, PMS1, MSH2, and MSH6 (24). This leads to a rare childhood cancer predisposition syndrome with recessive inheritance. The spectrum of CMMRD tumors is broad and CDMMR patients possess a high risk of multiple cancers, including hematological, brain, and intestinal tumors (25).

Lynch syndrome (LS) is an autosomal dominant disorder resulting from:

- constitutional germline mutations, affecting the DNA MMR genes MLH1, MSH2, MSH6, and PMS2
- constitutional MLH1 methylation
- 3’ end truncating EPCAM deletion, causing allele-specific epigenetic silencing of the neighboring DNA mismatch repair gene MSH2 and subsequent hypermethylation of its CpG island promoter in tissues expressing EPCAM (26). In this context cancers can arise in the colo-rectum, endometrium, ovary, stomach, small bowel, gallbladder, hepatobiliary tract, pancreas, renal pelvis and/or ureter, bladder, kidney, brain, or prostate. Generally, the MLH1 variant is correlated with the highest risk of colorectal cancer, while the MSH2 variant is correlated with the highest risk of other cancers. ECs occurring in this setting represent 3%–5% of cases and often arise in younger women (45–55 years). EC is the index cancer in slightly more than 50% of cases.

Also, a substantial proportion (25%–30%) of non-LS endometrial carcinomas (sporadic ECs) have dMMR (27). Most are due to:

- sporadic somatic biallelic hypermethylation of the MLH1 gene promoter
- two somatic mismatch repair gene mutations
- one somatic mutation with LOH of the other allele
- alternatively, secondary epigenetic silencing of MSH6 is observed after neoadjuvant radio-chemotherapeutic treatments (28, 29).

### Clinical and Pathological Associated Features

The MSI/hypermutated group is characterized by a high mutational rate (18 × 10^−6^ mutations per megabase) and included both low-grade and high-grade EEC.

Clinically, regarding patients with dMMR ECs, their prognosis seems to consistently remain intermediate. In particular, in early-stage, low-grade EECs, which usually have a good prognosis, dMMR appears as a risk factor for relapses (30, 31). In high-grade EECs, generally characterized by an intermediate prognosis between low-grade EEC and non-endometrioid carcinomas, dMMR is similarly associated to an intermediate prognosis (32). In the dMMR group of EEC, both LVSI and deep myometrial invasion were found as independent prognostic factors, while a high FIGO grade was not (33). DMMR EECs with MLH1 promoter methylation seem to have a worse prognosis than dMMR EEC with the mutation of MMR genes (33), offering a possible substratification of the dMMR group.

In non-endometrioid carcinomas, which have a poor prognosis, dMMR is a favorable prognostic factor (34–38). So, it is possible to consider the DMMR group as an intermediate-risk group regardless of the histotype. An exception could be represented by undifferentiated/dedifferentiated EC (UEC/DEC), in which a loss of SWI/SNF proteins expression seems to be associated with aggressive behavior even in the case of a dMMR signature

Moreover, dMMR ECs are unlikely to respond to conservative hormonal treatment and show a high likelihood of lymphovascular space invasion justifying a sentinel or other nodal procedure, and have not shown significant survival benefit by a chemotherapeutical approach. On the other hand, they seem to respond well to radiotherapy.

From a pathological point of view, dMMR/MSI ECs are more frequently characterized by the following distinctive gross and histological features:

- Lower uterine segment origin
- Significant peritumoral and intratumoral infiltrating lymphocytes (≥40 TIL/10HPFs), with more CD8+, CD45RO+, and PD1+ T cells at the invasive margin in LS—endometrial cancers compared with sporadic dMMR endometrial neoplasms (39).
- Synchronous ovarian cancer (clear cell or endometrioid variants)
- Higher grade with undifferentiated component
- Phenotypic heterogeneity (defined as two morphologically distinct tumor populations)
- > LVSI
- > Deep myoinvasion (higher prevalence of MSH2 MSH6 loss in MELF+ EECs has also been reported) (40)
- > Stage

Thus, morphology can significantly improve the efficacy of dMMR/MSI detection, because pathologists, on the basis of peculiar histopathological features often associated with a genetic profile, could identify “sentinel case”, with high suspicion for LS.

However, Mills et al. showed that more than half of the LS-related endometrial tumors (58%) did not have the classical pathological MSI tumor features (41). On this wave, pathological features are not always specific except for an endometroid histology and studies show contradicting results. In conclusion, to support a morphologic suspicion, in order to detect LS-related endometrial cancers, a universal screening is recommended.

### Reasons for MMR Testing in EC

MMR screening/MSI testing has several important clinical implications:

- screening for LS: it is estimated that one in 250–300 people are affected by LS and EC is often the first or sentinel cancer and can precede subsequent cancers such as colorectal carcinoma by approximately a decade. The identification of LS in a family allows surveillance and preventive measures in order to reduce the mortality from subsequent LS-related cancers.
- histomolecular diagnosis of EC: the TCGA classification and the recent ESGO/ESTRO/ESP guidelines require MMR testing of all cases for identification of the hypermutated dMMR/MSI EC group, with important management implications;
- predictive testing: dMMR tumors are eligible for targeted treatment with immune checkpoint inhibitors and are also characterized by overexpression of PD-1/PD-L1 (42).

In particular, recently, anti-PD1 antibody dostarlimab (Jemperli) has been approved by EMA and FDA to treat patients with dMMR or MSI-high (MSI-H) recurrent or advanced EC that has progressed after platinum-based chemotherapy.

### Laboratory Diagnostic Tests

Detection of dMMR can be carried out by IHC for the MMR proteins or through MSI testing. The two methods have comparable sensitivity and show approximately 96% concordance.

MMR protein IHC is the highly accurate surrogate of MSI molecular testing in EC. It is the recommended test to assess the presence or absence of MLH1, PMS2, MSH2, and MSH6 (alone or in combination with MSI testing) (**Supplementary Figure**). IHC analysis is usually preferred over MSI testing, for the following advantages:

- it is cheaper
- it has a lower turnaround time
- it is easily available
- it needs only a limited amount of tissue (i.e., 4 tissue slides)
- it is amenable to IHC external quality assurance schemes
- it allows correlation with morphology
- it enables the rapid identification of the defective protein, thus guiding downstream testing

Although a combination of only two antibodies MSH6 and PMS2 may reduce the cost without a significant decrease in the diagnostic accuracy as proposed by some paper, we discourage the use of a two-antibody (i.e., PMS2 and MSH6) approach (21). MMR protein expression in normal tissues is seen as nuclear staining, with uniform or variable intensity, according to the proliferative cell activity. In cancer cells, generally characterized by higher proliferation rates than normal tissue, the staining intensity is typically stronger and higher than that seen in the background stroma, normal glands, or inflammatory cells (internal control). Nuclear expression of all 4 mismatch repair proteins on IHC suggests MSS. Loss of nuclear staining for any of the proteins with an internal positive control indicates MSI.

There are 4 typical abnormal MMR IHC patterns:

- loss of both MLH1 and PMS2; this occurs in MLH1 deficiency (mutation/methylation)
- loss of both MSH2 and MSH6; this occurs in MSH2 deficiency (mutation)
- isolated loss of MSH6; this occurs in MSH6 somatic deficiency
- isolated loss of PMS2; this occurs in PMS2 somatic deficiency

It is important to note that MMR IHC loss with absence of MLH1 promoter methylation does not equate to LS, and only about half of the cases will be proven to have an inherited defect. The majority of these occur due to biallelic somatic loss of an MMR protein (27).

### Problems and Pitfalls in MMR IHC Interpretation

IHC can be considered valuable only in presence of a well-identifiable positive internal control (background stroma, normal glands, or inflammatory cells).

However, in some instances, a pathologist could run into the following problems or diagnostic pitfalls (43) (**Supplementary Table**):

- MMR IHC is very fixation-sensitive. Poorly fixed areas typically show negative staining in the absence of stromal staining, with a gradual decrease of intensity from positive areas. It is necessary that well-fixed areas are examined when reporting MMR IHC, to avoid erroneous interpretation. For this reason, MMR IHC should be carried out on better-preserved samples (biopsies or surgical samples). When the testing is performed in a preoperative setting, there is the added advantage that this molecular information is available at the time of EC diagnosis.

- Very weak/very focal expression may be seen in the presence of DMMR. As already stated, the expression of MMR proteins is generally strong and diffuse compared to the internal control, so that any deviation from this, including very weak/focal expression in the presence of an unequivocal positivity in stromal cells, should be noted and reported either as defective or equivocal/indeterminate. For example, weak focal/patchy immunoreactivity for MSH6 can be seen with MSH2 loss of expression/germline mutations. Repeating the staining on a different section or on biopsy specimen could solve some of these issues.
- Subclonal expression, defined as a focal loss of expression by tumor cells (at least 10% of the tumoral area, to assign tumor to the dMMR group); in order to distinguish it from variable expression as a result of a fixation artifact, normal staining must be seen in the internal control in the area showing expression loss in tumor cells. The cutoff of 10% is suggested to avoid reporting this pattern in cases where it is extremely focal and of unlikely clinical significance. Subclonal expression (generally subclonal MSH6 loss) is believed to occur as an acquired secondary (non-germline) defect, a “passenger mutation” arising from an underlying dMMR (in particular MLH1 defect) or in the presence of pathogenic POLE mutations. Subclonal MSH6 loss is sometimes accompanied by variable expression of MSH2. When subclonal MSH6 loss occurs with another defect, the reporting terminology should be as for the underlying defect. When subclonal MSH6 loss occurs as an isolated abnormality, this should be reported as abnormal as it may indicate an underlying germline abnormality, most likely in a gene other than MSH6. The behavior of these cases with regard to prognosis and responses to treatment is unknown.

- Punctate nuclear expression in a low proportion of MLH1 loss cases. This pattern should be reported as loss of expression and is thought to be a technical artifact, seen with the MLH1 (M1) clone (Roche Diagnostics)
- Cytoplasmic/dot-like/membranous staining should be reported as abnormal. It is possibly related to technical reasons
- Geographical loss of MLH1 and PMS2 due to heterogeneous hypermethylation within the tumor
- Loss of 3 or all proteins (total loss)
- Discordance or heterogeneity between MMR IHC and MSI:
  - MSS with loss of MMR protein expression due to MLH1 promoter hypermethylation or somatic MMR variants
  - MSI with retained/proficient MMR protein expression (6%–7%) due to POLE variants or determined by catalytically inactive mutated MMR proteins (missense mutation) that retain their antigenic integrity

MSI testing is based on PCR amplification of microsatellite markers. The pentaplex panel of five poly-A mononucleotide repeats (BAT-25, BAT-26, NR-21, NR-24, and NR-27) is the recommended panel given its higher sensitivity and specificity (44). Historically, loss of stability in 1 of the five microsatellite markers was defined as MSI-low and loss of stability in ≥2 as MSI-high. Currently, MSI-low tumors should be included within MSS tumors (45).

In selected experienced centers, NGS can represent an alternative novel molecular test to assess MSI, especially in non-Lynch-associated tumors (46), with the main advantage consisting in coupling MSI analysis with the determination of tumor mutational burden (TMB), along with other possibly targetable alterations.

Subsequent testing for MLH1 hypermethylation in order to identify a MLH1/PMS2-negative tumor as sporadic and (in the absence of a germline mutation) somatic mutations should be used to further evaluate the risk of subsequent cancers

## p53 IHC as Surrogate of TP53 Mutation

### Clinical Definition and Therapeutical Implications

p53 protein was discovered in 1979 as a 53-kilodalton protein from SV40 transformed cells (47) and was recognized as a tumor suppressor protein in 1992 (48). Mainly acting as a transcriptional factor, it plays important roles in the regulation of cell proliferation, DNA repair, apoptosis, genomic stability, senescence, and metabolic homeostasis (49). When DNA is damaged, p53 induces the expression of p21, a cyclin-dependent kinase (CDK) inhibitor that suppresses cyclin–CDK complexes, resulting in cell cycle arrest in the G1 phase, in this way allowing DNA repair before replication at S1 (50, 51). If the cells cannot repair the DNA damage, p53 induces apoptosis by activating apoptosis signal genes, such as BAX, PUMA, Noxa, and PERP (52). Loss of p53 function allows abnormal cell dysregulated proliferation, and this phenomenon is closely associated with carcinogenesis. Dysfunction of p53 has been observed in many malignant tumors (53, 54), mainly due to the inactivation of p53 protein by binding proteins or TP53 mutations. Majority of p53 mutations in cancer are missense mutations, leading to the expression of full-length mutant p53 (p53abn) protein. Many tumor-associated p53abn proteins not only lose the tumor-suppressive function of wild-type p53 but also gain new activities to promote tumorigenesis and tumoral progression, termed gain of function.

In endometrial cancer, the copy-number high/serous-like group is characterized by low mutational rate (2.3 × 10^−6^ mutation per megabase) but extensive SCNA somatic copy-number alteration (SCNA), with TP53 mutation in 90% of cases (17).The copy-number high/serous like group mainly includes high-grade tumors, in particular SECs and UCSs. Although accounting for only 15% of all ECs, it is responsible for 50%–70% of endometrial cancer mortality, showing a poor outcome in all histotypes and justifying the classification of all carcinomas with a p53abn phenotype in the high-risk category irrespectively of other factors (30, 38, 55, 56). However, prognostic differences between different histotypes may still exist, due to the simultaneity of other unfavorable clinicopathological factors (57).

For instance, p53abn SC might be more aggressive than p53abn EEC and p53abn CC-EC but less aggressive than p53abn UCS (58), but until now, it is not well known if these differences can justify different types of clinical treatment. Moreover, we have to remember that also a small percentage (2%–5%) of low-grade EECs shares TP53 mutations, exhibiting a mutation-type immunoreactivity.

Recent evidence has shown improved survival outcomes with the addition of chemotherapy compared with radiation alone in p53abn ECs. Targeted therapy for p53abn ECs also exists with a proportion of p53abn ECs known to have homologous recombination deficiency (HRD), DNA damage, high PARP-1 expression, or human epidermal growth factor 2 (HER2) overexpression/amplification (see later in the text). In fact, PARP inhibitors and anti-HER-2 targeted therapy with trastuzumab have shown promising results in these tumors (59).

### Immunohistochemical Interpretation

Only a validated optimized laboratory protocol including appropriate “high expressor” positive control (HGSC), low-expressing (e.g., tonsil), and negative control tissues (colon) together with a correct pathological interpretation of p53 immunohistochemical staining are fundamental to achieve high diagnostic accuracy in predicting the presence of TP53 mutation and to obtain high interlaboratory concordance (60).

A 4-tier system (61, 62), recommended for p53 IHC interpretation, in the presence of a well-recognizable positive internal (non-neoplastic cells such as lymphocytes, fibroblasts, or endothelial cells) consists of:

1. abnormal/aberrant/mutation type—overexpression: diffuse and uniformly strong nuclear expression of p53 in virtually 100% of tumor cell nuclei in a well-fixed case and at least 75% of tumor cell nuclei in a less well-fixed case, generally due to non-synonymous missense mutations
2. abnormal/aberrant/mutation type—complete absence, due to nonsense mutations
3. abnormal/aberrant/mutation type—cytoplasmic expression (with a variable nuclear staining), generally associated to loss-of-function mutations disrupting the nuclear localization domain of p53
4. normal—wild type: nuclear p53 expression is observable in <80% of cells and/or with variable intensity, in absence of TP53 mutation, but we have to keep in mind that in about 5% of cases, a mutator genotype due to some splice site mutations or truncating mutations can also result in detectable, nonfunctional p53 protein with a wild-type staining pattern.

Cases without a positive internal control must be reported as uninterpretable.

### Problems and Pitfalls in p53 IHC Interpretation

- Wild-type different grading: depending on the cellular differentiation and the proliferative activity, the normal wild-type pattern can show a wide range of staining, from only very few scattered tumor cell nuclei positive (low wild-type) to the majority of nuclei being positive (high wild-type).
- Heterogeneous p53 expression/subclonal TP53 mutation: a proportion of G3 EEC or ambiguous carcinomas with an ultramutated or hypermutated genotype (either POLEmut or DMMR) can acquire a TP53 mutation later in the tumoral course, developing a subclonal TP53 mutation that may result in heterogenous p53 expression, with a combination of normal wild-type and abnormal patterns (overexpression and/or ‘null’ phenotype and/or cytoplasmic staining). In these cases (generally defined multiple classifiers, as DMMR/p53abn ECs—64%; POLEmut/p53abn ECs—31%), an experienced gynecopathologist together with an optimized immunohistochemical protocol are necessary to assess such patterns (63), differentiating them from mixed cancers or from the possibility of a “wild-type variability” and “p53 mosaic patterns”. We retain that in case the morphologic features suggestive of POLE mutations (see later in the text) are present and p53 staining pattern is abnormal, it may be useful to repeat the stain on a different tumor section.
- Mosaic patterns of p53: generally due to preanalytical factors (delayed fixation with antigen degradation) or to rare molecular alterations (splice site mutation or low allelic frequency of TP53 mutation in some tumor areas) that determine variable intensity of the staining (some staining strong, some moderate, some weak, few negative).
- Nonspecific nuclear blush due to technical artifacts, could be misinterpreted as wild type in cases of true complete absence.
- Nonspecific cytoplasmic blush due to technical artifacts, could be misinterpreted as p53abn. It represents an equivocal blush, which may be ignored.
- Mixed abnormal patterns (complete absence and overexpression) indicating either different clonal origin or tumor progression with acquisition of a different type of TP53 mutation.
- p53 overexpression in a subset of EECs (without an underlying mutation) can be explained by dysregulation of other factors such as ERβ and MDM2.

## No Specific Molecular Profile Surrogate of the Copy Number Low/Endometrioid Group

The copy-number-low/endometrioid group is characterized by low mutational rate (2.9 × 10^-6^ mutations per megabase), with no specific molecular profile (NMSP; no MSI or POLE mutations) and with a low degree of somatic copy-number alteration (SCNA, no p53 mutations) (17). Consistently lacking of molecular signatures, the NSMP also lacks a univocal prognostic significance.

Generally, the NSMP group shows a good-to-intermediate prognosis in low-grade EEC, an intermediate outcome in high-grade EEC, and poor prognosis in non-endometrioid carcinomas (30, 32, 35, 37, 38, 56).

The NSMP group may further be stratified based on histological (i.e., tumor grade and histotype, LVSI, depth of myometrial invasion), immunohistochemical (i.e., L1CAM expression), or molecular (i.e., CTNNB1 mutation) features (64).

### Beta-Catenin as a Surrogate of CTNNB1 Mutation?

Beta-catenin is a multifunctional protein expressed on the surface of epithelial cells, acting as a structural component of the E-cadherin-related cell adhesion system (65). This molecule has a crucial role in maintenance of epithelial stability by regulating cell growth and intercellular adhesion. Beta-catenin also plays other important functional roles, including control of cell polarization, differentiation, “stemness,” and cell motility (66). At the cellular level, beta-catenin is the key mediator of the Wnt canonical pathway (67, 68). Constitutive activation of the Wnt signaling is a major etiological factor for many cancers (69, 70).

In the resting state of Wnt signaling, beta-catenin is phosphorylated by glycogen synthase kinase 3ββ (GSK3-β) within a protein complex including casein kinase 1, adenomatous polyposis coli (APC), and Axin. Phosphorylated beta-catenin is immediately degraded *via* the ubiquitin–proteasome pathway. In the active state, WNT binds to Frizzled (Fz), activating (Dsh), which inhibits the activity of GSK3-β, resulting in dephosphorylation and stabilization of beta-catenin, in this way enabling accumulation within the nucleus. A stable beta-catenin translates WNT signal into the transient transcription of a TCF/LEF (T-cell factor/lymphocyte enhancer factor) gene program, governing cell fate, proliferation, and other processes in several types of cancer (71, 72). Stabilized molecule could be considered a cause of genomic instability, promoting tumor development and aggressiveness. Numerous studies have shown that CTNNB1, the gene of beta-catenin, together with APC and Axin are frequently mutated in different types of human cancers and that the nuclear accumulation of beta-catenin could be considered the final step of constitutive activation of WNT signaling (73).

In particular, in EC, it has been described that a subset of low-grade, early-stage EECs (about 50% of NSMP EECs and almost 20% of all ECs) can harbor CTNNB1 exon 3 mutations, and when this happens, prognosis in terms of OS and recurrence-free survival is worse (31, 70, 74), overall intermediate, although their frequent clinic–pathological characteristics are commonly associated with lower risk of recurrence (younger age, squamous differentiation, low TILs, less incidence of deep myometrial invasion, and less incidence of LVSI, with a low number of other concurrent mutations, such as KRAS and FGFR2 mutation) (64).

Nuclear accumulation of beta-catenin, well detectable by immunohistochemistry, has been assessed as a possible surrogate for sequencing to identify CTNNB1-mutant cases, resulting in conflicting data, varying from an overall good agreement between protein nuclear staining and CTNNB1 status (70, 75, 76) to high discordance.

It seems that nuclear accumulation of beta-catenin in EEC implies the presence of CTNNB1 mutation, but not *vice versa* (76, 77). Furthermore, no standardized criteria for interpretation of beta-catenin immunostaining have been defined in order to consider its expression normal vs. aberrant. It seems that even a minimal percentage of beta-catenin positive nuclei should be considered significant, while only a nuclear expression being at least moderate should be considered of diagnostic significance.

Finally, in most cases, nuclear beta-catenin in EEC is limited to the morular metaplasia foci, not present in all CTNNB1-mutant cases and not clearly correlated to prognosis (76–79).

## Multiple Molecular Classifiers and Interpretation Issues

Multiple classifier EC is defined as a tumor harboring more than one molecular classifying feature:

- dMMR/p53abn
- POLE mut/p53abn
- dMMR/POLE mut/p53abn
- dMMR/POLE mut

The simultaneous presence of two or three molecular signatures is encountered in 3% of ECs and outcomes correspond to those predicted by the driver molecular subtype; in particular, the POLEmut signature, when characterized by a pathogenic status, prevails over the other signatures, conferring a good prognosis regardless of MMR and p53 status, while the dMMR signature prevails over the p53abn signature (18). These findings support:

- the classification of tumors with a pathogenic POLE EDM and dMMR and/or p53abn as single classifier POLEmut ECs
- the classification of tumors with a wild-type POLE and dMMR and/or p53abn as single classifier DMMR
- the classification of tumors with a dMMR and p53abn as single classifier DMMR ECs

In this way, in the context of dMMR EC or POLEmut ECs, passenger secondary events such as the occurrence of TP53 mutation, do not affect biological behavior and should not prompt intensified treatment.

## Open Questions and Perspectives from the Consensus Panel

### Hormonal Receptor Status

#### Clinical Definition and Immunohistochemical Interpretation

ER and PR belong to the superfamily of steroid receptors, regulating the hormonal activity in the different phases of the endometrial cycle (80). Binding to its ligand, the hormonal receptor leads to translocation of the ligand–receptor complex to the nucleus where receptor dimers bind specific hormone-responsive DNA elements of target genes (81). In the endometrium, estrogen determines proliferation, while progesterone inhibits estrogen-induced endometrial proliferation (82).

The nuclear presence of ER and PR in tumor tissue is routinely evaluated by IHC in different organs, and also in pathological–neoplastic conditions. Immunohistochemical loss of ER and PR expression in tumor tissue is associated with a higher risk of node metastases and poor prognosis (reduced disease-free survival and disease-specific survival) and with poor response to hormonal therapy (63, 83–86). Scoring systems, based on percentage alone or on combinations of percentages and intensity of staining are used frequently (87–89), although the percentage score has been retained more relevant than staining intensity scores as confirmed by recent studies (90, 91).

However, the cutoff value for ER and PR positivity with the strongest prognostic value for clinical outcome in EC is still unclear (83, 90, 92). Over time, different cutoff values have been proposed: 1% or 10% of positive tumor nuclei, or a staining-intensity index of 3 (on a 0–9 scale) (87, 93–97).

Recently, the ENITEC collaboration study has proposed an EC-specific classification for ER and PR expression categorized into three groups: a high-risk group–low HR expressing (ER/PR expression: 0%–10%), with unfavorable outcome (5-year DSS 75.9%–83.3%); an intermediate-risk group (ER/PR expression: 20%–80%) with intermediate outcome (5-year DSS 93.0%–93.9%); and a low-risk group–high HR expressing (ER/PR expression: 90%–100%) with a favorable outcome (5-year DSS 97.8%–100%). In this study, at the first cutoff value of 10%, ER had a higher specificity, indicating that ER ≤10% is more able to identify high-risk cases; at the cutoff value of 80%, PR had a higher sensitivity, suggesting that PR is more able to identify a low-risk population (98). Contradictory results regarding the prognostic value of ER/PR expression within TGCA molecular subgroups have been reported, due to application of multiple cutoff values for ER/PR expression (64, 99)

### L1CAM as a Tool to Further Clinically Stratify NMSP EECs?

L1, also known as L1CAM, is a transmembrane cell surface glycoprotein member of the L1 protein family, encoded by the L1CAM gene, sited in the long arm of the X chromosome in Xq28 position. This protein, of 200-220 kDa, is a neuronal cell adhesion molecule with important roles in cell migration, adhesion, and neuronal differentiation (100). It is normally expressed in neuronal and non-neuronal cells and also overexpressed in multiple tumor cells, making them more aggressive and chemo-resistant. In this way, this upregulation is associated with tumor progression, acquisition of EMT phenotype, and metastasis, causing poor prognosis (101–104).

Regarding EC, some authors have found significant association between L1CAM (>10%) and p53-mutant, both related to risk recurrence for EC patients (105). According to another recent work, L1CAM detection has been used as a surrogate of LVSI, and so lymph node involvement, with the advantage of also a possible preoperative evaluation (106).

Moreover, it has been demonstrated that L1CAM stratifies patients with NSMP: high L1CAM immunohistochemical expression (>10%) was associated to highly aggressive tumors, characterized by poor differentiation, NE histology, and worse prognosis independently from age of patients (higher risk of distant recurrences and pelvic lymph-node involvement) (107, 108).

### PDL1

#### Definition and Therapeutic Implications

Programmed death-ligand 1 (PD-L1, CD247, or B7-H1) is one of the ligands of the programmed cell death 1 (PD-1) receptor, a dominant negative regulator of antitumor T-cell effector function (56). It is induced by inflammation and expressed by tumor microenvironment and tumor cells (20). The blockade of the PD-1–PD-L1 binding by the use of selected specific antibodies has become a novel therapeutic tool in tumors overexpressing PD-L1 or in tumors with activated T-cell immunoresponse and high tumoral mutation burden. Anti-PD-1/PD-L1 therapies determine T-cell proliferation and infiltration into the tumoral area, promoting an increased cytotoxic T-cell response, leading to an evident tumor response (109, 110).

Regarding EC, since the high mutational load and abundance in neoantigens, the POLEmut and DMMR groups are considered optimal candidates to respond to anti-PD-1/PDL1 treatment (immune checkpoint blockade therapy) and the assessment of PDL-1 expression may be reasonable in these tumors (42, 111).

The recent ESGO/ESTRO/ESP guidelines of EC have approved dMMR/MSI as the selection criteria for the second-line standard of care anti-PD-1/PD-L1 immune therapy with pembrolizumab. In MMR stable ECs, a combined second-line therapy consisting of pembrolizumab+lenvatinib, a multi-tyrosine kinase inhibitor, could be considered as a possible option (7).

#### Clinical and Pathological Associated Features

Regarding EC, considerable variations in PD-L1 positivity frequencies (from 0.9% to 44.3%) have been reported (16, 112–123). Moreover different PD-L1 expression profiles between molecular subclasses, histologic subtypes, and tumoral stage have been described, with the POLE mutant, the dMMR, the non-endometrioid types, and the advanced endometrial cancers displaying the highest PD-L1 levels in TCs and ICs, and CPS (111).

Regarding the association between PDL1 and prognosis, data are still unclear. Some authors showed that high PD-L1 in tumor cells (TCs) was associated with better OS and longer treatment-free interval (TFI) after primary chemotherapy in recurrent cases, whereas high PD-L1 in tumor-infiltrating immune cells (TICs) was associated with worse OS as well as MSI (124). On the other hand, a recent meta-analysis showed that in EC, PD-L1 expression seems to be not associated with poor prognosis (OS and PFS), while it is positively correlated with poor differentiation and advanced tumor stage (125).

### HER2

Her2 (human epidermal growth factor receptor 2) or HER2/neu, also known as receptor tyrosine-protein kinase erbB-2, or CD340, or ERBB2, is an oncogenic protein encoded by the ERBB2 proto-oncogene, located at the long arm of human chromosome 17 (17q12) (126). It is a member of the human epidermal growth factor receptor (HER/EGFR/ERBB) family that is involved in the activation of different signaling pathways, such as mitogen-activated protein kinase (MAPK), phosphoinositide 3-kinase (PI3K/Akt), protein kinase C (PKC), and signal transducer and activator of transcription (STAT), generally promoting cell proliferation and inhibiting the apoptosis process (127). Normal tissues have a low complement of HER2 membrane protein. Its amplification/overexpression plays an important role in the development and progression of 15%–30% of breast cancers and 7%–34% of gastric cancers, conferring worse biological behavior and clinical outcome (128), although the protein has become an important biomarker for targeted molecular therapy (129).

Over-expression also occurs in approximately 25% to 30% of endometrial serous carcinomas, with over 50% of HER2-positive tumors showing marked intratumoral HER2 immunohistochemical or genic heterogeneity (130).

With particular regard to endometrial serous carcinoma, nowadays there is a growing demand for new-targeted therapies for this aggressive tumor type, characterized by the highest recurrence rate and considered responsible for 40% of endometrial cancer mortality, with an overall 5-year survival rate of only 45% (131–133).

Recently, promising clinical results have been obtained from a multi-institutional, randomized phase 2 clinical trial, demonstrating that the addition of trastuzumab to standard chemotherapy resulted in significant improvement in progression-free and overall survival in HER2-positive advanced stage and recurrent endometrial serous carcinoma (59). This therapeutical approach limited to serous tumor subtype, either pure or as a component of mixed EC, was endorsed by the National Comprehensive Cancer Network (NCCN) Uterine Neoplasm Guidelines (134). On this wave, the pathologic testing and scoring of tumoral HER2 protein expression and gene amplification may be considered a critical part of patient selection for this type of targeted therapy.

**Figure 4:**
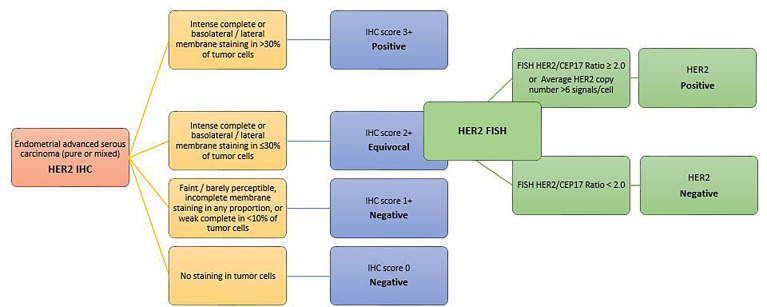
HER2 scoring method in endometrial carcinomas A set of HER2 testing algorithm and scoring criteria have been proposed for routine pathological evaluation of endometrial serous carcinoma. Scoring categories have been classified according to the modified 2007 American Society of Clinical Oncology/College of American Pathologists [ASCO/CAP] breast criteria.

It is well known that distinct characteristics of HER2 protein expression and gene amplification can be observed in different tumors of different organ systems (135, 136). Thus, similarly to breast and gastric cancer, based on evidence from the recent successful clinical trial and 2 comprehensive pre-trial pathologic studies (137, 138), a new set of HER2 testing algorithm and scoring criteria have been proposed for routine pathological evaluation of endometrial serous carcinoma. Scoring categories have been classified as follows (139–141) (**Figure 4**):

- 0 HER2 NEGATIVE: No staining in tumor cells
- 1+ HER2 NEGATIVE: Faint/barely perceptible, incomplete membrane staining in any proportion, or weak complete staining in <10% of tumor cells
- 2+ HER2 EQUIVOCAL: Strong complete or basolateral/lateral membrane staining in ≤30%, or weak to moderate complete or basolateral/lateral staining in ≥10% of tumor cells
- 3+ HER2 POSITIVE: Strong complete or basolateral/lateral membrane staining in >30% of tumor cells (modified 2007 American Society of Clinical Oncology/College of American Pathologists [ASCO/CAP] breast criteria).

FISH should be performed only on tumors with a 2+ immunohistochemical score on a large tumor area in direct correlation with the HER2 immunostained slide, and a HER2/CEP17 ratio of ≥2.0 can be considered amplified. However, until more data become available to define whether HER2 amplification could be considered equal or superior to INC in predicting clinical therapeutical response, tumors with HER2/CEP17 ratio <2 and average HER2 copy number ≥6.0/nucleus should also be included in the HER2 amplified category.

Future studies are necessary to resolve a number of practical pathological issues.

Currently, there are only limited data available on optimal specimen type (biopsy versus hysterectomy) for HER2 testing in uterine serous cancer.

Recently, discordant HER2 status has also been observed between primary and metastatic endometrial tumors, in particular in heterogeneous cancers (142, 143).

Furthermore, another laboratory practical problem is represented by the optimal HER2 testing algorithm (IHC as primary test vs. FISH as reflex test), depending on the correlation between test type and therapeutic response.

Similarly to breast and gastric cancers, further clinicopathologic studies will be essential to define the pathological concept and the clinical impact of intratumoral heterogeneity and its correlation to therapeutic response (144). Only fewer data are available regarding HER2 positivity rates in early-stage endometrial serous carcinoma: only a recent study showed a statistically significant association between HER2 positivity and poor prognosis in terms of DFS and OS (145).

Finally, there is a strong clinical interest in expanding targeted HER2 therapy to other high-grade EC in the future, due to the recently identified similarities in HER2 protein expression/gene amplification between endometrial serous carcinoma and endometrial carcinosarcomas (146–148).

### Preoperative Setting

Nowadays, an intriguing challenge is represented by the application and validation of TCGA classification in small diagnostic biopsies/endometrial curetting, in order to decide the adequate surgical/clinical approach. Recent studies suggest highly concordant results between diagnostic biopsies and hysterectomy specimens, in particular for MMR loss, MSI high, and p53 wild and aberrant types, in contrast to moderate levels of agreement reported for the classical histomorphological parameters (grade, histotype). In this way, in the setting of an adequate technical and medical training, with well-experienced professional figures and well-defined laboratory protocols, molecular classification preoperatively applied seems to provide earlier and more reliable prognostic information to guide clinical management (149).

The bioptical specimen, being immediately placed in formalin compared with hysterectomy specimens that may sit for several hours before processing, allows a better tissue fixation and a superior antigen preservation consequently may assure a more reproducible and adequate biological characterization (150) in cases in which endometrium on surgical sample is sub-optimally preserved for immunostains.

Other possible applications of molecular categorization on bioptical samples could consider the role of MSI status in predicting resistance to progestins for early-stage EECs and the overall unfavorable prognostic significance of MMR deficiency in conservatively treated EECs (151).

Nevertheless, the potential limit derived from the adoption of the molecular TGCA classification to small biopsies in daily practice could derive from the occurrence of intratumoral heterogeneity, with possible molecular discrepancies between the initial endometrial biopsy and the hysterectomy specimen.

### Optimal Tissue Handling and Sectioning Requirements for Interlaboratory Reproducibility

The increasing clinical demand for a histomolecular approach in classifying ECs should prompt pathology laboratories to implement specific protocols for specimen handling and fixation. We must be aware that molecular and IHC testing can be affected by:

- Pre-analytical variables: specimen collection and handling, tissue fixation (uniformity, time, and type) and protocol of processing
- Analytical variables: choice of immunohistochemistry protocol, reagent variability, antigen retrieval technique, and technician training/expertise
- Post-analytical variables: evaluation of positive/negative controls, morphological correlations, diagnostic and prognostic significance, correlation with other data, interpretation and reporting of results, and experience/expertise of the pathologist.

The goal consists in ensuring reproducibility, obtaining high quality of stained sections with minimal inter-observer variability in the diagnostic report and in promoting inter-laboratory standardization, which are all synonyms of quality assurance. We retain that it can be achieved only by the application of automated systems (e.g., Ventana and Dako systems) and FDA-approved kits or validated laboratory platforms and tests (152, 153).

In particular:

- Time from tissue acquisition to fixation should be as short as possible (cold ischemia time of less than 1 h)
- Samples for molecular/IHC testing should be fixed in 10% neutral buffered formalin for 6–72 h for both endometrial biopsy and hysterectomy specimens
- Samples should be sliced at 5- to 10-mm intervals after appropriate gross inspection and margin designation and placed in a sufficient volume of neutral buffered formalin. Any exceptions to this process must be included in the report.
- Sections should ideally not be used for IHC testing if cut >6 weeks earlier; this may vary with primary fixation or storage conditions
- Optimal internal validation procedure of the test must be performed before test is offered

## Conclusions

For the correct assignment of ECs to one of the 4 molecular subgroups, a correct interpretation of molecular and immunohistochemical data is fundamental.

Currently, there is no surrogate markers for POLE mutation, and targeted sequencing for the common mutations or whole genome or panel testing must be used.

In order to support a morphologic suspicion and/or to detect LS-related endometrial cancers, a universal screening is recommended for MMR testing in EC, with important management and therapeutical implications.

Similarly to MMR deficiency detection by IHC, a validated optimized laboratory protocol together with a correct pathological interpretation of p53 immunohistochemical staining are fundamental to achieve high diagnostic accuracy in predicting the presence of TP53 mutation, in this way selecting candidate patients for targeted therapy with PARP inhibitors.

Considering the NSMP group, it may be stratified based on histological (i.e., tumor grade and histotype, LVSI, and depth of myometrial invasion), immunohistochemical (i.e., L1CAM expression), or molecular (i.e., CTNNB1 mutation) features. Up-to-date contradictory results regarding the prognostic value of ER/PR expression within all the TGCA molecular subgroups have been reported.

Considerable variations in PD-L1 positivity frequencies have been reported and data regarding the association between PDL1 and prognosis are still unclear.

Based on the modified 2007 ASCO/CAP guidelines for breast cancer, a new set of HER2 testing algorithm and scoring criteria have been proposed for routine pathological evaluation of endometrial serous carcinoma, helping to select patients eligible for antiHER2 therapies.

Considering all these advances in the EC molecular landscape, we retain that, in the personalized medicine era, although molecular classification of ECs is going to open a new interesting scenario by introducing novel molecularly driven treatment choices for EC, pathology is still crucial for clinical management. In this new context, molecular classifiers should be combined with clinical risk groups and pathological parameters in an integrated histo-molecular approach, able to better discriminate outcomes for different patients.

## Author Contributions

All authors contributed to the study conception and design. AS, FI, AP, MF, and EB performed the literature search. FF, FC, GT, and AS performed data analysis. AS, GZ, and FI drafted and/or critically revised the work. All authors contributed to the article and approved the submitted version.

## Conflict of Interest

The authors declare that the research was conducted in the absence of any commercial or financial relationships that could be construed as a potential conflict of interest.

## Publisher’s Note

All claims expressed in this article are solely those of the authors and do not necessarily represent those of their affiliated organizations, or those of the publisher, the editors and the reviewers. Any product that may be evaluated in this article, or claim that may be made by its manufacturer, is not guaranteed or endorsed by the publisher.

## Supplementary Material

Supplementary Figure Flow chart for Lynch Syndrome triage, by the use of IHC for 4 MMR proteins as a first choice test
