# Supplementary figures and images for: Current Prognostic and Predictive Biomarkers for Endometrial Cancer in Clinical Practice: Recommendations/Proposal from the Italian Study Group
Source: Front Oncol. 2022 Apr 8;12:805613. doi: 10.3389/fonc.2022.805613 (PMC9024340; doi:10.3389/fonc.2022.805613)

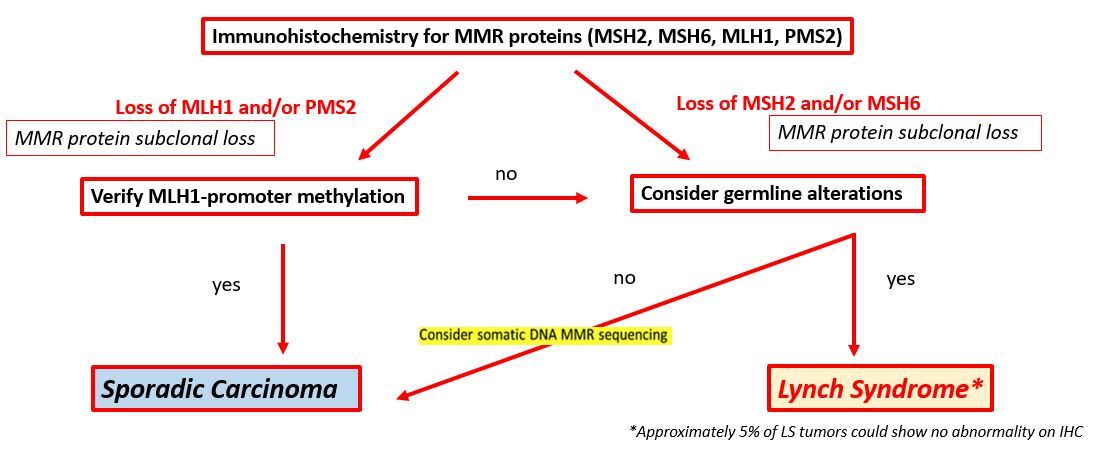

Supplement: Supplementary Figure — Flow chart for Lynch Syndrome triage, by the use of IHC for 4 MMR proteins as a first choice test [file Image_1.jpeg]
