# Supplementary material for: Current Prognostic and Predictive Biomarkers for Endometrial Cancer in Clinical Practice: Recommendations/Proposal from the Italian Study Group
Source: Front Oncol. 2022 Apr 8;12:805613. doi: 10.3389/fonc.2022.805613 (PMC9024340; doi:10.3389/fonc.2022.805613)
Supplement: Supplementary file 2 [file Table_1.docx]

**Table 1. Recommended terminology for reporting MMR IHC and/or MLH1 promoter methylation status**

| MLH1 | PMS2 | MSH2 | MSH6 | Recommended Report | Conclusion | Referral to Genetics |
| --- | --- | --- | --- | --- | --- | --- |
| + | + | + | + | MMR stable immunophenotype | NO IHC evidence of MMRd | NO |
| + | + | + | - | *MMR instable immunophenotype*  MSH6 loss | MMRd is associated with Lynch and related syndromes | YES |
| + | - | + | + | *MMR instable immunophenotype*  PMS2 loss | MMRd is suggestive of Lynch and related syndromes | YES |
| + | + | - | + | *MMR instable immunophenotype* MSH2 loss | MMRd is probably due to Lynch or related syndromes. | YES |
| - | - | + | + | *MMR instable immunophenotype*  MLH1 loss  No MLH1 Promoter hypermethylation | MMRd is probably due to Lynch or related syndromes. | YES |
| - | - | + | + | MMR instable immunophenotype  MLH1 loss  MLH1 Promoter hypermethylation | MMRd is almost certainly sporadic | NO |
| - | - | + | + | *MMR instable immunophenotype*  MLH1 loss  MLH1 Promoter hypermethylation not tested | This pattern is likely to be sporadic, although it  is possible that this MMRd is due to Lynch or related syndromes | YES  (or testing MLH1 hypermetilation) |
| Subclonal loss | Sub-  clonal loss | + | + | *MMR instable immunophenotype* Subclonal MLH1 loss | This pattern is likely to be sporadic, although it  is possible that this MMRd is due to Lynch or related syndromes | Testing for MLH1 Promoter hypermethylation is recommended. |
| - | - | + | Subclonal loss | MMR instable immunophenotype | MMRd may be associated with Lynch and related syndromes | YES |
